# His-Tag-Aptamer-Liposome-Conjugates as a Multipurpose Tool for Recombinant Protein Screening and Protein-Based Bioassays

**DOI:** 10.1021/acs.analchem.6c00320

**Published:** 2026-04-24

**Authors:** Pia Schraml, Florian K. Blaser, Simon Streif, Nico Dreymann, Axel Duerkop, Antje J. Baeumner

**Affiliations:** † 9147 University of Regensburg, Faculty of Chemistry and Pharmacy, Institute of Analytical Chemistry, Chemo- and Biosensors, Universitätsstraße 31, 93053, Regensburg, Germany; ‡ Fraunhofer Institute for Cell Therapy and Immunology, Branch Bioanalytics and Bioprocesses (IZI-BB), Am Mühlenberg 13, 14476, Potsdam-Golm, Germany

## Abstract

Liposome-aptamer conjugates are powerful tools for bioanalysis and drug delivery by taking advantage of aptamer binding specificity and liposome encapsulation capabilities. Here, we developed multipurpose conjugates that specifically and effectively bind to his-tags and demonstrated their capability as screening tool for his-tagged proteins and as signaling tool for bioassays. Using the poly histidine-tag (his-tag) binding aptamer NDM1-H14–01 (his-Apt), we studied aptamer-liposome immobilization with special focus on retaining aptamer functionality and liposome integrity. This involved various strategies for modifying liposomes with aptamers, i.e., cholesterol-functionalized aptamers were inserted into the liposome pre- or post-synthesis, amine-modified aptamers were covalently coupled to COOH-groups on the liposome surface, and biotinylated aptamers were incubated with streptavidin-modified liposomes, respectively. Extensive characterization of liposome and aptamer properties, as well as conjugate functionality via heterogeneous binding assays, proved post-insertion and biotinylated aptamers successful with a stability of at least 4 months at 4 °C, whereas the other methods destabilized the aptamer structure. The successful conjugates were further optimized to (i) serve as a tool for easy detection and screening of his-tag accessibility in recombinant proteins, which can replace common metal-based formats (ii) enable efficient site-directed immobilization of proteins onto liposomes while omitting the need of additional protein modifications, and (iii) demonstrate that aptamers can function as a reliable secondary recognition element for protein-modified liposomes, e.g., as internal control system. Consequently, these highly functional and stable his-Apt-liposome conjugates represent a versatile platform technology for bioassays, which can substitute labor- and cost-intensive measurements, as well as improve assay control and reproducibility.

## Graphical abstract

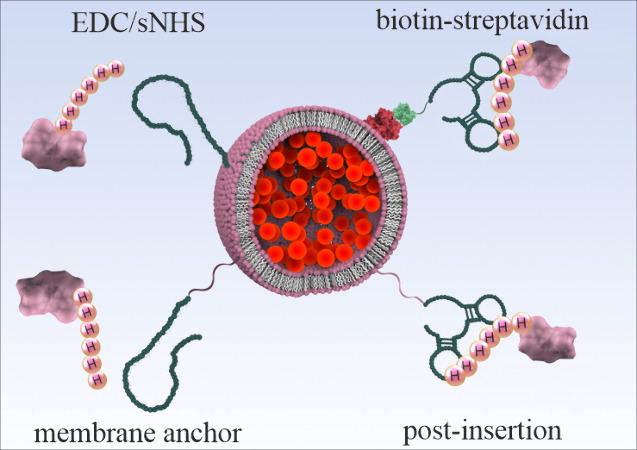

The use of aptamers as affinity biorecognition elements for a broad range of analytes, from small molecules through proteins to whole cells, has been of growing interest since their first description in 1990 by Tuerk and Gold^1^ and Ellington and Szostak.^2^ Similar to antibodies, aptamers possess the ability to bind to protein targets with high affinity and specificity, often exhibiting K_d_-values in the nanomolar to picomolar range. The secondary structure motifs, as well as the 3-dimensional folding of aptamers, play a pivotal role in their binding ability.^3^ Predominantly determined by Watson–Crick base pairing, aptamers often fold into structural motifs like hairpin stem-loops, kissing-loop complexes, and pseudoknots, but also non-canonical structures like wobble pairs and G-quadruplexes are possible.^5,6,3,4^ Hairpin stem-loops are the most widespread motifs with different shapes, including multibranched loops, internal loops, or a bulge alignment.^7^ Overall, the stability of the different motifs can vary and be influenced by small cations and buffer compositions.^9,8,11,10^ In particular, divalent cations, like Mg^2+^ or Ca^2+^, are important for the formation of complex tertiary structures.^12^ General advantages of aptamers described in literature include low batch-to-batch variations, non-toxicity, and non-significant immunogenicity, as well as their adaptability to variable, even non-physiological conditions, with reversible denaturation.^14,13,15^ Conclusively, chemical modifications can easily be introduced to diversify the aptamer’s properties and enable the application of numerous bioconjugation strategies for the development of aptamer-drug, aptamer-protein, and aptamer-particle conjugates.^16,18,4,17^ Here, conjugation of aptamers to liposomes is being investigated, as both find application in drug delivery, and in bioassay and biosensor development.^19–21^

Liposomes are defined as self-assembled spherical vesicles spontaneously formed by phospholipids dissolved in aqueous media and consist of one or more concentric lipid bilayers enclosing a hydrophilic cavity capable of encapsulating hydrophilic molecules.^22^ The polar headgroups are oriented toward the core and the surrounding solution, while the nonpolar lipid chains face each other, resulting in an amphiphilic bilayer.^19,23^ During liposome synthesis, a wide variety of encapsulants can be incorporated into the hydrophilic cavity.^19,24^ For analytical applications, one of the main focuses lies on signal amplification based on the high surface area and large internal volume of the liposomes, combined with high encapsulation efficiencies and sensitivity of the reporter molecules.^25,19^ Often used examples represent fluorescent detection, e.g., using carboxyfluorescein and sulforhodamine B (SRB),^29,27,28,24,26^ but also colorimetric,^30^ electrochemical,^22^ as well as chemi- and bioluminescent formats.^32,31^ These encapsulants enable the application of liposomes in various bioassays in homogeneous and heterogeneous formats.^34,33,24,21,26^

To combine all these advantages through the formation of liposome-aptamer conjugates, versatile covalent and non-covalent bioconjugation strategies can be applied depending on the reactive groups available within the lipid headgroups.^36,37,35^ A widespread reaction type is the formation of a covalent amide bond by the linkage of a carboxylic acid group and a primary amine through 1-ethyl-3-(3-dimethylaminpropyl) carbodiimide (EDC).^37,39,38^ Generally, covalent coupling strategies share the advantages of high stability, specificity, and the possibility of long-term interactions. However, these reactions also present the disadvantages of irreversibility and potential interference with the ligand’s biological activity, e.g., due to perturbations on the aptamer conformation at the nanoparticle surface.^40,36,37^ These drawbacks can be circumvented by non-covalent bioconjugation methods based on specific binding pairs, with biotin and streptavidin displaying the most established example.^24,38,35^ Further special reaction-free bioconjugation strategies with liposomes include conjugation during synthesis, called the membrane anchor method, or after synthesis, called post-insertion, which both rely on lipid-modified ligands.^44,41–43^ For the membrane anchor method, the ligand is directly incorporated into the lipid bilayer during the synthesis process, which facilitates the purification of the liposomal formulation; however, resulting in a major drawback, the ligands can be affected by exposure to organic solvents.^45^ Alternatively, the post-insertion method is applied after synthesis, where ligand-modified lipids are incubated with preformed liposomes.^46,47,4^ The ease of this process and the gentle conditions allow for flexible conjugation and reaching of high insertion efficiencies through fine-tuning of incubation temperature and duration.^46,44,4^

Due to the importance of recombinant proteins in various fields, such as biological or biopharmaceutical research,^49,48^ and the high abundance of the poly histidine-tag (his-tag) as an affinity tag within recombinant proteins,^50^ aptamers directed against the his-tag are of high scientific interest. Among those, several publications have presented the preparation of his-tag-aptamer modified magnetic beads or resins as a tool for recombinant protein purification, displaying a potential alternative to the established immobilized metal affinity chromatography (IMAC) format.^52,51,55,53,54^ These aptamer-based systems stand out for distinct advantages: One substantial property is their superior direct applicability to crude samples and supernatants owing to the high target specificity and lower levels of nonspecific binding, which enables facile screening e.g., of successful expression and binding studies.^51,54^ Thereby, end point purity can be achieved in single-step formats while omitting the need for preceding time-consuming optimization procedures associated with Ni-NTA-based methods.^52,51^ In addition, the versatility of aptamers opens the possibility of imidazole-independent elution without requiring competitive agents, consequently improving downstream applicability of the targets.^56,51^ A critical step for all those purification techniques, however, lies in the proper accessibility of the his-tag on the proteins and consequently a strong affinity toward the separating agent, which is too often not the case and leads to low purification yields. Specifically, this is fundamentally influenced by tag location, protein folding, or involvement in intramolecular interactions, and can thereby impact purification efficiency and subsequent applicability.^59–61,58,62,57^ Screening studies of his-tag accessibility are thereby so far mostly designed with metal-based formats, e.g., for investigating suitable tag positions for T4 lysozyme,^58^ or for monitoring the effect of glycosylation on α-chymotrypsin.^63^

In this work, the his-tag aptamers NDM1-H14–01­(-FR) (his-Apt­(-FR)) are presented as an innovative, versatile tool for bioassays, based on aptamer-liposome conjugates, whose preparation was carefully optimized through comparing common synthesis strategies. The application as a detection system for recombinant proteins, as well as a screening platform for his-tag accessibility in different proteins, is performed in a rapid, easy, and reliable heterogeneous binding assay format, exemplarily conducted for the recombinant proteins HSA, RBD, and ACE2. Additionally, the conjugates serve as a basis for site-directed, non-covalent coupling, utilizing the aptamer as an anchor for the modification of liposomes with recombinant proteins, which can prevent interferences with the protein’s functionality and provide maximum flexibility.^64^ Lastly, the implementation into liposome-based bioassays as an internal control system through dual-modified liposomes coupled with the biorecognition element of interest simultaneously to the his-Apt-FR aptamer enables the enhancement of precision, repeatability, and reliability in the hitherto underexplored field of internal control setups.^65^

## Experimental Section

### Chemicals and Consumables

All chemicals used were of analytical reagent grade. *n*-octyl-β-d-glucopyranoside (OG) (CN23, ≥98%), 4-(2-hydroxyethyl)-piperazine-1-ethanesulfonic acid (HEPES) (HN87, ≥99.5%), 2-(*N*-morpholino) ethanesulfonic acid (MES) (4259, ≥99%), d-(+)-sucrose, calcium chloride (CaCl_2_), potassium chloride (KCl), sodium chloride (NaCl), tris­(hydroxymethyl)-aminomethane (TRIS), and regenerated cellulose dialysis membrane Spectra/PorⓇ 4 (MWCO: 12–14 kDa) (2718.1) were purchased from Carl Roth (Karlsruhe, Germany); cholesterol from sheep wool (C8667, ≥ 99%), SephadexⓇ G-50, sulforhodamine B (SRB) (230162), *N*-hydroxysulfosuccinimide sodium salt (sNHS) (≥98%), TWEENⓇ 20, from Sigma-Aldrich (Steinheim, Germany); 1,2-dipalmitoyl-*sn*-glycero-3-phosphoethanolamine-*N*-(glutaryl) (sodium salt) (*N*-glutaryl-DPPE), 1,2-dipalmitoyl-*sn*-glycero-3-phosphocholine (DPPC), 1,2-dipalmitoyl-*sn*-glycero-3-phospho-(1′-*rac*-glycerol) (sodium salt) (DPPG), and the extruder set from Avanti Polar Lipids (Alabaster, USA); polycarbonate membranes (0.2 μm, 0.4 and 1.0 μm pore size, Ø 19 mm) from Whatman (Dassel, Germany); Magnesium chloride hexahydrate (MgCl_2_), disodium hydrogen phosphate (Na_2_HPO4), potassium dihydrogen phosphate (KH_2_PO_4_), l-lysine monohydrochloride, sodium hydroxide (NaOH), hydrochloric acid (HCl), NHS-biotin (203112) and bovine serum albumin fraction V (BSA) from Merck (Darmstadt, Germany); polyclonal anti-RBD IgG antibody produced in rabbit (PA5–114451), polyclonal antihuman serum albumin antibody produced in chicken (PA1-72054), 1-ethyl-3-(3-(dimethylamino)­propyl) carbodiimide hydrochloride (EDC) (PG82079), Pierce protein concentrators (MWCO: 10 kDa), and Nunc MaxiSorp black high-binding microplates (96-well, flat bottom) from Thermo Scientific (Waltham, USA); black microtiter plates (96-well, flat, black bottom) from Brand (Wertheim, Germany); black streptavidin plates (nonproteic blocking, MTBSTDF4-SB75/100/W) from Biomat (Ala, Italy); chloroform, methanol, nitric acid (70%, HNO_3_), dimethyl sulfoxide (DMSO), and Spectra-PorⓇ Float-A-LyzerⓇ G2 (1 mL, MWCO: 1000 kDa) from Fisher Scientific (Schwerte, Germany); phosphorus standard for ICP measurements from Bernd Kraft (Duisburg, Germany); Protein LoBindⓇ Tubes from EppendorfⓇ (Hamburg, Germany); DNALowBind micro tubes from Sarstedt (Nuembrecht, Germany); human serum albumin protein his tag (his-HSA, HSA-H5220) from Acro Biosystems (Basel, Switzerland); SARS-CoV-2 RBD (Variant of Concern Alpha B1.1.7), angiotensin-converting enzyme 2 (ACE2), and ACE2-biotin were provided by Mikrogen (Neuried, Germany) and Microcoat (Bernried am Starnberger See, Germany); Lysogen broth (LB) from Alfa Aesar (Haverhill, USA); *Escherichia coli* K12 (*E. coli*) from the DSMZ-German Collection of Microorganisms and Cell Cultures; aptamers 5′-NH2-C6-NDM1-H14–01-FR and 5′-Cholesteryl-NDM1-H14–01-FR from Metabion (Planegg, Germany); aptamers 5′-NH2-C6-NDM1-H14–01, 5′-biotin-NDM1-H14–01-FR, and 5′-biotin-NDM1-H14–01 from Biomers GmbH (Ulm, Germany); aptamer 5′-Cy5-NDM1-H14–01 from Integrated DNA Technologies (Coralville, USA); MST control aptamer 5′-Cy5-Con1 from Iba Lifesciences (Goettingen, Germany); Aptamer sequence information (5′-3′) were provided by the group of Dr. Marcus Menger (Fraunhofer Institute of Cell Therapy and Immunology, Branch Bioanalytics and Bioprocesses, AG Functional Nucleic Acids – Aptamers) as described by Sabrowski et al.^66^: NDM1-H14–01-FR (his-Apt-FR): CGTCATAG­GTACCAT­GGGGGA­CTGCTC­GGGATT­GCGGATT­CATG;

NDM1-H14-01 (his-Apt): GTATCT­GGTGGT­CTATGGCGT­CATAGGTA­CCATGGG­GGACTGCTC­GGGATTG­CGGAT­TCATGGCA­TAGA­CGAC­GAAGAAC;

Con1: GGGAATT­CGAGCTCG­GTACCGGCT­GCTTTG­CTGCAGAT­TTGTGGG­TGGGTGGG­TGGTGATCTG­CAGGCATGC­AAGCTTGG.

### Buffer Solutions

All buffer solutions were prepared using double-distilled water, and the pH values were adjusted using NaOH or HCl. Phosphate buffered saline (PBS) consisted of 137 mM NaCl, 2.7 mM KCl, 10 mM Na_2_HPO_4,_ and 1.8 mM KH_2_PO_4_ with a pH of 7.4. For phosphate buffered saline TWEENⓇ 20 (PBS-T) additional 0.1 w/v% TWEENⓇ 20 was added to PBS. HEPES saline sucrose buffer (HSS) was prepared with 200 mM sucrose, 200 mM NaCl, 10 mM HEPES, and 0.01 w/v% NaN_3,_ and a pH value of 7.5 was set. The HEPES aptamer binding buffer (HABB) consisted of 10 mM HEPES_,_ 5 mM MgCl_2_ · 6H_2_O, 140 mM NaCl, 1 mM CaCl_2_, and 1 mM KCl with a pH of 7.5. For the phosphate aptamer binding buffer (PABB), 10 mM Na_2_HPO_4_ · 2H_2_O and 1.8 mM KH_2_PO_4_ were included instead of HEPES, with a pH of 7.4, and for the TRIS aptamer binding buffer (TABB), 20 mM TRIS with the pH value adjusted to 7.4. For the TRIS binding buffer (T-BP), an additional 0.05 v/v% TWEENⓇ 20 was added to TABB. For the high osmolality aptamer binding buffers (h.o.HABB, h.o.PABB, h.o.TABB), 200 mM sucrose was added to each of the low osmolality aptamer binding buffers (HABB, PABB, TABB). High osmolality MES buffer (h.o.MES) contained 0.05 M MES, 200 mM sucrose, and 200 mM NaCl with a pH of 5.5. Lysogen broth medium (LB medium) was prepared by dissolving 10 mg of LB in 500 mL double-distilled water.

### Aptamer Folding and Binding Characterization

Before initial use, the aptamers were always prepared using the standard protocol, as described by Sabrowski et al.,^66^ for ensuring proper folding: The aptamers were diluted to 30 μM in the aptamer binding buffers and heated to 92 °C for 3 min, then left at RT until fully cooled. Calculations of aptamer folding and secondary structure motifs were conducted by the UNAFold Web site.^67^ The surrounding temperature and the sodium and magnesium concentrations in the buffer samples were used as a base for the calculations.

### Circular Dichroism Measurements

The aptamers were diluted to 5 μM and measured at 22 °C in a wavelength range of 320 to 215 nm, with a bandwidth of 1 nm, a data pitch of 0.5 nm, a scan speed of 100 nm/min, and an accumulation of n = 10. The background was measured before each sample under the same conditions and automatically subtracted from the sample spectrum. Molar ellipticity was converted using the mean molar mass of the four monophosphate nucleobases. All measurements were conducted with a JASCO spectropolarimeter type J-710 (Jasco Deutschland GmbH, Pfungstadt) in Hellma quartz cells Suprasil with a path length of 1 mm.

### Microscale Thermophoresis

Microscale thermophoresis (MST) experiments were performed on a Monolith NT.115 (NanoTemper Technologies, Munich, Germany) using standard capillaries. Measurements were conducted at an MST power of 40% and excitation power ranging from 20% to 40%. 5′-Cyanine5-labeled (Cy5) aptamers were refolded in T-BP buffer as previously described and diluted to a concentration of 20 nM. Serial dilutions of his-HSA 0.27–8900 nM, his-RBD 0.31–10,000 nM, and his-ACE2 0.14–4600 nM were prepared in T-BP buffer. After combining equal volumes of refolded aptamer and protein dilution, the mixtures were incubated for 30 min before measurement. The recorded fluorescence was normalized to the ΔF_Norm_ in per mill and fitted utilizing the K_d_ formula derived from the law of mass action by MO Affinity Analysis software (NanoTemper Technologies GmbH, Munich, Germany) version 2.3.^69,68^ All measurements were performed as duplicates or triplicates.

### Liposome Synthesis and Characterization

Liposomes were synthesized according to the reverse phase evaporation method as described by Streif et al.^26^ For this liposome composition, containing the encapsulant with a concentration of 150 mM SRB, an encapsulation efficiency of 2.6% can be attributed, corresponding to ∼ 85000 molecules of SRB per liposome, as determined by Streif et al.^64^ For aptamer-modified liposomes, an additional 0.05 mol % 5′-Cholesteryl-NDM1-H14–01-FR (Chol-his-Apt-FR) was added to the lipid mixture. To determine the total lipid (tL) concentration, the phosphorus concentration was measured with a Spectroblue Inductively Coupled Plasma Optical Emission Spectrometer (ICP-OES) (SPECTRO Analytical Instruments GmbH, Kleve, Germany) at λ = 177.495 nm. Calibration was conducted by dilution of the phosphorus standard solution in 0.5 M HNO_3_ to 1, 5, 10, 50, and 100 μM tL, and before each measurement with the 0 μM and 100 μM solution. The liposome samples were diluted 1:100 for unmodified liposomes or 1:60 for modified liposomes in 0.5 M HNO_3_. For additional determination of the total lipid concentration c(tL) for aptamer-containing liposomes, the sample volume $V_{2}$ was determined after the modification process, and the concentration calculated based on the amount of substance added. Based on this calculated liposome concentration, the aptamer coupling efficiency was evaluated by subtracting the liposome concentration from the total phosphorus concentration measured by ICP, revealing the proportion of the aptamers. The hydrodynamic diameter, polydispersity, and surface charge of unmodified and modified liposomes were determined by dynamic light scattering measurements (DLS) with a Malvern Zetasizer Nano-ZS (Malvern Panalytical, Malvern, United Kingdom). For hydrodynamic diameter (Z-average) and polydispersity index (PdI) determination, liposomes were diluted to 50 μM tL (unmodified) or 25 μM tL (modified) in HSS or high osmolality aptamer binding buffers (dispersant refractive index $n_{D}^{20}$ = 1.34 (all buffers), dielectric constant ε = 78.5 (all buffers), viscosity η = 1.1185 cP (HSS), η = 1.0631 cP (h.o.HABB)). 500 μL of each solution was measured in PMMA semimicro cuvettes (Brand, Wertheim, Germany) with three measurement cycles containing 13 individual measurements each at 25 °C after a 15-s equilibration time. For zeta-potential, liposomes were diluted to 50 μM tL (unmodified) or 12.5 μM tL (modified) in the respective buffers (HSS, h.o.HABB) with 800 μL of the solutions filled in folded capillary zeta cells (Malvern Panalytical, Malvern, United Kingdom). After 60 s equilibration at 25 °C, four measurement cycles consisting of 30 individual measurements were conducted. To determine the maximum fluorescence and unlyzed fluorescence of unmodified and modified liposomes, liposomes were diluted to 50 μM tL (unmodified) or 5 μM tL (modified) in HSS or h.o.HABB and pipetted into a black Brand 96-well microplate in quadruplets (100 μL per well), with and without addition of 30 mM OG (lyzed liposomes). The fluorescence was measured three consecutive times with a BioTek SYNERGY neo2 fluorescence reader (Agilent Technologies, Santa Clara, USA) (λ_E__xc_ = 560 nm and (λ_Em_ = 585 nm, bandwidth 5 (50 μM tL) or 10 (5 μM tL), gain 100). For the calculation of the unlyzed fluorescence, the fluorescence of the unlyzed liposomes was divided by the maximum fluorescence.

### Liposome Modification

Calculation of the aptamer-liposome surface coverage based on the molar fraction of aptamers coupled to the liposomes was executed according to the method described by Streif et al.^64^ Covalent coupling via EDC/sNHS was conducted using carboxyl groups on the outer liposome surface. Liposomes, EDC, and sNHS (1:100:180 ratio of carboxy-groups:EDC:sNHS), with both coupling reagents dissolved to 10 mg/mL in h.o.MES buffer, were incubated for 1 h at RT and 300 rpm in DNALowBind micro tubes or protein LoBindⓇ Eppendorf tubes, with subsequent addition of the calculated percentage of the amine-modified aptamers 5′-NH2-C6-NDM1-H14-01-FR (NH2-his-Apt-FR) or 5′-NH2-C6-NDM1-H14–01 (NH2-his-Apt) or proteins, related to the amount of total lipids, with further incubation for 1.5 h at RT and 300 rpm. For streptavidin (stav)-modified liposomes, lysine-HCl was added (final concentration of 10 mM) for quenching, and the solution was incubated for further 15 min at RT and 300 rpm. For purification from excess reagents, either size exclusion chromatography with Sephadex G-50 (MWCO: 30 kDa, Sigma) or dialysis with Spectra-PorⓇ Float-A-LyzerⓇ G2 (MWCO: 1000 kDa, Sigma) for 24 h with two buffer exchanges against HSS or h.o.HABB was conducted. For the modification with the post-insertion method (PI), Chol-his-Apt-FR and liposomes were incubated for 2 h at varying temperatures from RT to 55 °C in DNALowBind micro tubes. For purification from excess aptamers and encapsulant, size exclusion chromatography with SephadexⓇ G-50 (MWCO: 30 kDa, Sigma-Aldrich) against h.o.HABB was conducted. Modified liposomes were stored in DNALowBind micro tubes at 4 °C. For the non-covalent interaction-based modification, liposomes modified with 1.38 mol % stav were mixed with the biotin-modified aptamers 5′-biotin-NDM1-H14–01-FR (biotin-his-Apt-FR) or 5′-biotin-NDM1-H14–01 (biotin-his-Apt). This step was implemented in heterogeneous binding assays. Stav-liposomes, diluted to 1 μM tL in h.o.HABB, and biotin-aptamers, diluted to 1 nM - 20 nM, were mixed and simultaneously added to the microtiter plate (total volume of 100 μL per well) and incubated for 2 h at RT and 300 rpm. All other steps were performed according to the general heterogeneous binding assay procedure.

### Heterogeneous Binding Assays

Heterogeneous binding assays were conducted in triplicates for all samples. For the general procedure, Nunc MaxiSorp high-binding microplates were coated with his-tag or non-his-tag bearing proteins (0 μg/mL, 1 μg/mL, or 2 μg/mL in PBS, 100 μL per well) by incubation overnight at 4 °C. The wells were emptied and then blocked with BSA (1 w/v% in PBS-T, 150 μL per well), with incubation for 1 h at RT and 300 rpm. Afterward, the wells were washed two times with PBS-T and three times with 150 μL of either HSS, h.o.HABB, h.o.PABB, or h.o.TABB per well. Liposomes were then diluted to 1 μM tL in one of the respective buffers or cell lysate (0 vol %, 10 vol %, 100 vol %) and incubated for 2 h at RT and 300 rpm (100 μL per well). After that, the wells were again washed three times with the previous buffer, before the liposomes were lyzed by the addition of 30 mM OG in double-distilled water (100 μL per well). The fluorescence was measured three consecutive times with a BioTek SYNERGY neo2 fluorescence reader (λ_E__xc_ = 560 nm and (λ_Em_ = 585 nm, bandwidth 10, gain 150). For the sandwich assays, either ACE2-biotin (0 μg/mL or 2 μg/mL in PBS, 100 μL per well) was incubated in a Biomat streptavidin microplate for 1 h at RT and 300 rpm, or the anti-HSA antibody (PA1–72054, 0 μg/mL or 5 μg/mL in PBS, 100 μL per well) in a Nunc MaxiSorp high-binding microplate overnight at 4 °C. His-tag proteins diluted in h.o.HABB were added to the post-inserted aptamer-liposome solutions, and the samples were pre-incubated in solution for 30 min. For the neutralization assay, the liposomes (1 μM total lipids) and anti-RBD antibodies (0.001–5 μg/mL) were diluted in h.o.HABB and preincubated for 2 h at RT and 300 rpm, before addition to the Biomat streptavidin microtiter plate, coated with ACE2-biotin or HSA-biotin. All further experimental procedures followed the general protocol. HSA-biotin was prepared through incubation of his-HSA with 10 equiv of NHS-biotin per protein for 2 h at RT with subsequent centrifugal filtration. If not stated differently, h.o.HABB was used as the standard liposome buffer for all binding assays.

### Cell Lysate Preparation

Cultures were prepared by inoculating 10 mL of LB medium with one colony of *E. coli* and incubating overnight at 37 °C. The overnight cultures (OD_600 nm_ = 0.3 in 1:20 dilution) were centrifuged for 10 min at 5000 g and 4 °C, the supernatant removed, and the pellet resuspended in h.o.HABB. After repeating the washing procedure a further 2 times, lysis was performed via ultrasonication at a frequency of 20 kHz and an intensity of 320 W for 5 min with 30 s on- and off-intervals with the Bioruptor (Diagenode, Seraing, Belgium). Cell debris was removed by a final centrifugation step (15 min at 15000 g and 4 °C).

### Data Evaluation

All measurements were conducted multiple times, with the data presented as the average ± standard deviations. Fitting of measurement data was performed with Origin 2024, OriginLab Corporation (Northampton, USA). Dose–response curves were fitted with the “logistic” function according to $y=\frac{A_{1}-A_{2}}{1+{\left(\frac{x}{x_{0}}\right)}^{p}}+A_{2}$ , and EC50 values extracted. Normalized fluorescence refers to normalization of fluorescence intensities in binding assays to the respective liposome maximum fluorescence, using unmodified liposomes as reference value. Normalized binding for neutralization assays was calculated based on normalized fluorescence values, using the sample without addition of neutralizing agents as reference (negative control): $\mathrm{normalized}\;\mathrm{binding}\;\left(\%\right)=\left(\frac{\mathrm{norm.}\;\mathrm{fluor.}\;}{\mathrm{norm.}\;\mathrm{fluor.}\;\mathrm{neg.}\;\mathrm{control}}\right)\cdot 100$ . Outliers were generally identified via Q-test with 90% confidence or Grubbs-test with a significance level 0.1.

## Results and Discussion

Highly functional and long-term stable his-tag-aptamer-liposome conjugates are developed, to be applied as a universal tool with recombinant proteins, e.g., for screening of his-tag affinity, to function as ligand for conjugation of proteins onto liposomes, or to be implemented as an internal control system in bioassays. Studies were hence designed to generate stable and reliable aptamer-liposome conjugates while maintaining both of their functionalities.

### Determination of General Buffer Conditions for Functional Aptamer-Liposome Conjugates

For the development of aptamer-liposome conjugates, the identification of buffer conditions that promote liposome stability and aptamer functionality is necessary, as liposomes require appropriate osmolality and low divalent cation concentrations, whereas aptamers require buffers used during their selection process. For this study, the NDM1-H14-01 (his-Apt) and NDM1-H14-01-FR (his-Apt-FR) aptamers, directed against the poly histidine-tag (his-tag), were used as a model aptamer.^66^ The significantly shorter primer truncated his-Apt-FR (44 nucleotides, 13739 Da) should have comparable binding properties to the full-length his-Apt version (80 nucleotides, 24990 Da), as identified by surface plasmon resonance by Sabrowski et al.^66^ The secondary structures of both aptamer versions were calculated with the UNAFold server.^67^ The lowest Gibbs free energy structures (Figure S1) display two hairpin stem loops as most dominant motifs for both aptamer variants. For liposome assays, HEPES, TRIS, and phosphate buffers are most often used, adjusted to the required high osmolality needed to best maintain their integrity.^70–73^ The influence of these high osmolality aptamer binding buffers (h.o.ABB) on aptamer secondary and tertiary structures was hence studied using circular dichroism (CD) (Figure 1a). According to literature, for the calculated hairpin and B-form DNA structure motifs, two positive peaks in the range of 265 and 285 nm, as well as a negative peak in the range of 240 nm, were expected.^74,75,4^ H.o.HABB exactly resembled literature data, which suggests optimum folding in HEPES-based buffers. In h.o.TABB, the maxima, however, were slightly shifted toward each other, suggesting worse folding conditions yet with intact hairpin motifs. The spectra measured in h.o.PABB, in contrast, showed an altered curve shape, which corresponds to an unfolded B-DNA-like structure.^74^ This indicates that the phosphate-based buffers weaken the hairpin secondary structure motif. In fact, the structure change was confirmed when aptamer-liposome conjugates were synthesized with the post-insertion method for 1 mol % Chol-his-Apt-FR (described below in detail), and allowed to bind to his-tag HSA immobilized in a microtiter plate. Bound liposomes were quantified upon liposome lysis using fluorescence detection (Figure 1b). As expected, the aptamer liposomes bound best to immobilized his-HSA in h.o.HABB, and slightly worse for h.o.TABB. In the phosphate-based buffer, no binding could be achieved, confirming CD observations that indicated the unfolding of the relevant structural motifs in phosphate buffers.

**Figure 1:**
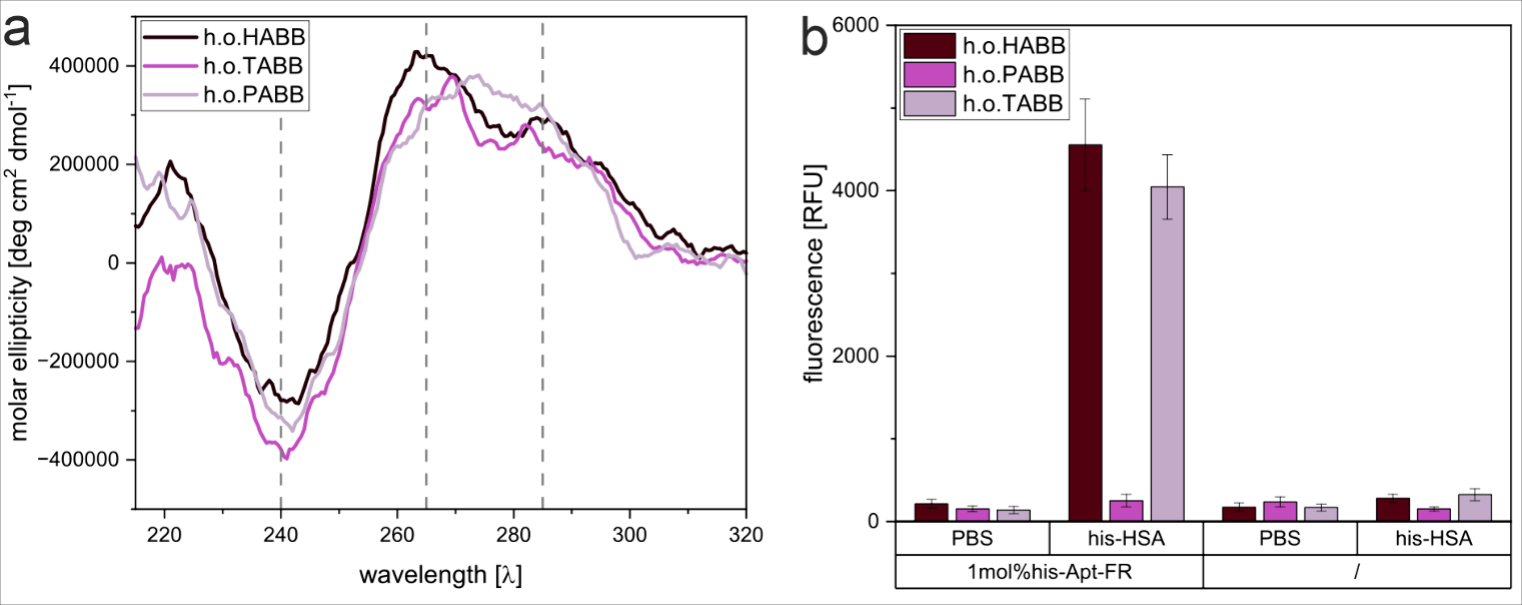
Examination of aptamer folding in different buffer systems through (a) circular dichroism spectra of the NH2-his-Apt-FR aptamer in either h.o.HABB, h.o.TABB, or h.o.PABB. Vertical lines indicate the expected maxima around 240, 265, and 285 nm. (b) binding assays depicting the fluorescence intensity of liposomes modified with 1 mol % Chol-his-Apt-FR through post-insertion or unmodified (/), immobilized in a Nunc MaxiSorp high-binding microplate coated with his-HSA (0 μg/mL or 1 μg/mL in PBS) in either h.o.HABB, h.o.TABB, or h.o.PABB. *n* = 3.

### Development of Aptamer Modified Liposomes

The coupling procedure exerts a significant impact on the stability and properties of functional aptamer-modified liposomes. In literature, common methods include covalent coupling by EDC/sNHS chemistry, non-covalent interaction through biotin–streptavidin, or the membrane anchor and post-insertion procedure. DNA modification of liposomes during synthesis by using cholesterol-modified oligonucleotides has long been established.^76^ We could demonstrate that this method is not generally applicable for aptamers without highly stabilizing secondary structures, i.e., adding cholesterol-tagged his-Apt to the lipid mixture prior to liposome preparation rendered only nonbinding aptamers. Specifically, liposome characterization proved aptamer presence; however, heterogeneous binding assays showed no binding to his-tagged proteins (Figure S2). Most likely, the extended contact with organic solvents led to a misfolded aptamer.^45^ Refolding could not be accomplished through extended dialysis in h.o.HABB, whereas the normal refolding protocol with heating to 92 °C could not be executed without resulting in full lysis of the liposomes due to surpassing the phase transition temperature of the lipids DPPC and DPPG (∼40 °C).^77^ Similarly, when amine-modified his-Apt­(-FR) was coupled to COOH-group-containing liposomes using standard EDC/NHS chemistry,^26^ liposomes were successfully tagged with the aptamer; however, no binding to his-tag proteins was obtained (Figure S3). Here, it is assumed that a perturbation of the aptamer folding was exerted through the coupling process or the covalent bond,^40,4^ regardless of the intrinsic spacer present in the full-length aptamer or the truncated aptamer version. Again, no refolding of the aptamer was possible via equilibration in h.o.HABB or via the normal heating procedure due to liposome instability. Hence, methods are required that maintain aptamer folding throughout the coupling procedure. The two strategies chosen included post-insertion of the aptamers,^46,4^ and biotinylation of the aptamers in combination with streptavidin-modified liposomes.^64^ For the post-insertion, lipid-modified aptamers were inserted into the readily synthesized liposomes, representing a highly gentle treatment for the aptamers.^46^ After initial successful insertion experiments, the preparation procedure was optimized with respect to insertion temperature and incubation time (Figure S4). To avoid encapsulant loss during the incubation step while maintaining high insertion efficiencies, temperatures between room temperature and 55 °C were studied. (Figure S4a-c). By reducing the incubation temperature, the liposome stability was significantly enhanced during the coupling process, while still efficient incorporation was guaranteed, revealing room temperature as the optimum condition in combination with incubation for 2 h (Figure S4b,c). The reliability and reproducibility of the method was demonstrated when varying the aptamer coverage on the liposomes. For example, for binding to his-HSA, immobilized in the microtiter plate, optimal coverage was found at 0.2 mol % Chol-his-Apt-FR of the total lipid (≙ ∼ 1250 aptamers per liposome), independent of the his-HSA amount present, which results in maximum binding of the aptamer-modified liposomes toward the proteins immobilized without steric hindrance observed for higher aptamer surface coverages (Figure S 4d-f).

As a fourth strategy, non-covalent coupling based on the interaction between biotin and streptavidin was tested with biotin-his-Apt-FR using liposomes coated with 1.38 mol % streptavidin, as optimized previously.^78^ Also with this method, highly functional aptamer-liposome conjugates could easily be generated. Incubation time and temperature were based on the modification of stav-liposomes with biotinylated RBD-variants, optimized by Streif et al.^64^ The reproducibility and reliability of this approach was demonstrated through varying aptamer coverage (Figure S5), where again, 0.2 mol % was found as optimal for this aptamer. In addition, the functionality of the two aptamer versions was very easily assessed (Figure 2). Specifically, it could be confirmed that the full-length and truncated aptamer versions his-Apt and his-Apt-FR, respectively, had highly comparable binding ability to immobilized his-tagged HSA (Figure 2a).

**Figure 2:**
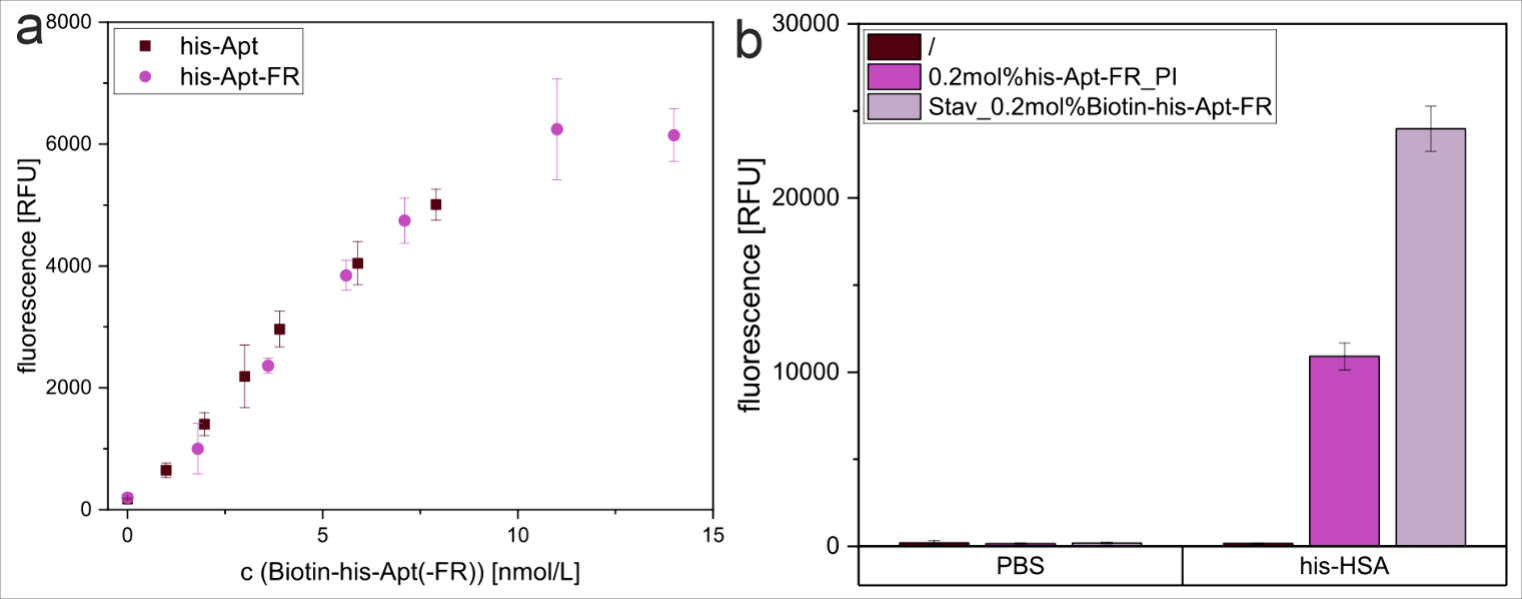
Comparison of conjugate functionality in binding assays, prepared with (a) the full-length or truncated aptamer through the biotin–streptavidin method, or (b) the truncated aptamer through the post-insertion or biotin–streptavidin method, showing the fluorescence intensity of liposomes immobilized together with (a) biotin-his-Apt (0 nM - 7.9 nM) or biotin-his-Apt-FR (0 nM – 14 nM) or (b) 0.2 mol % biotin-his-Apt-FR, Chol-his-Apt-FR_PI or unmodified liposomes (/), in a Nunc MaxiSorp high-binding microplate coated with his-HSA (2 μg/mL in PBS). *n* = 3.

Finally, using the his-Apt-FR conjugates, the two successful coupling strategies were directly compared in the heterogeneous binding assay (Figure 2b). About 65% higher intensity was obtained for the biotin–streptavidin format in a binding assay to his-HSA compared to the post-insertion approach. While the fluorescence intensity per total lipid of the streptavidin liposomes is 20% higher than that of the post-inserted liposomes, this accounts only for a part of the observed signal difference. It is therefore concluded that the streptavidin–biotin bridge functions as a spacer and provides the aptamer with a more favorable binding environment than the negatively charged liposome surface. In the future, a longer spacer between lipid and aptamer could be investigated.

### Storage Stability of Aptamer-Modified Liposomes

Since streptavidin-modified liposomes have been demonstrated to be stable for years at 4 °C,^78^ it is assumed that aptamer-liposome conjugate stability will be determined by the aptamer stability and hence relate to the presence of nucleases and stability of its secondary and tertiary structures. Post-inserted his-Apt-FR-modified liposomes were stored at 4 °C in h.o.HABB buffer at 660 μM tL. To monitor the preservation of the aptamer and liposome stability, binding assays with his-HSA (Figure 3a), and maximum and unlyzed fluorescence measurements (Figure 3b) were executed in regular intervals. In conclusion, the aptamer-modified liposomes were found to be stable for up to 16 weeks, whereas at 24 weeks, the aptamer binding ability and liposome stability were drastically reduced. We assume that this is due to DNA degradation over time. Since liposomes modified with DNA oligonucleotides prepared for DNA hybridization assays, and hence using the pre-synthesis coupling method, showed stability for up to 1 year in our lab,^80,79^ we will adapt to DNA nuclease-free methods in the future to further improve the aptamer-liposome conjugate stability.

**Figure 3:**
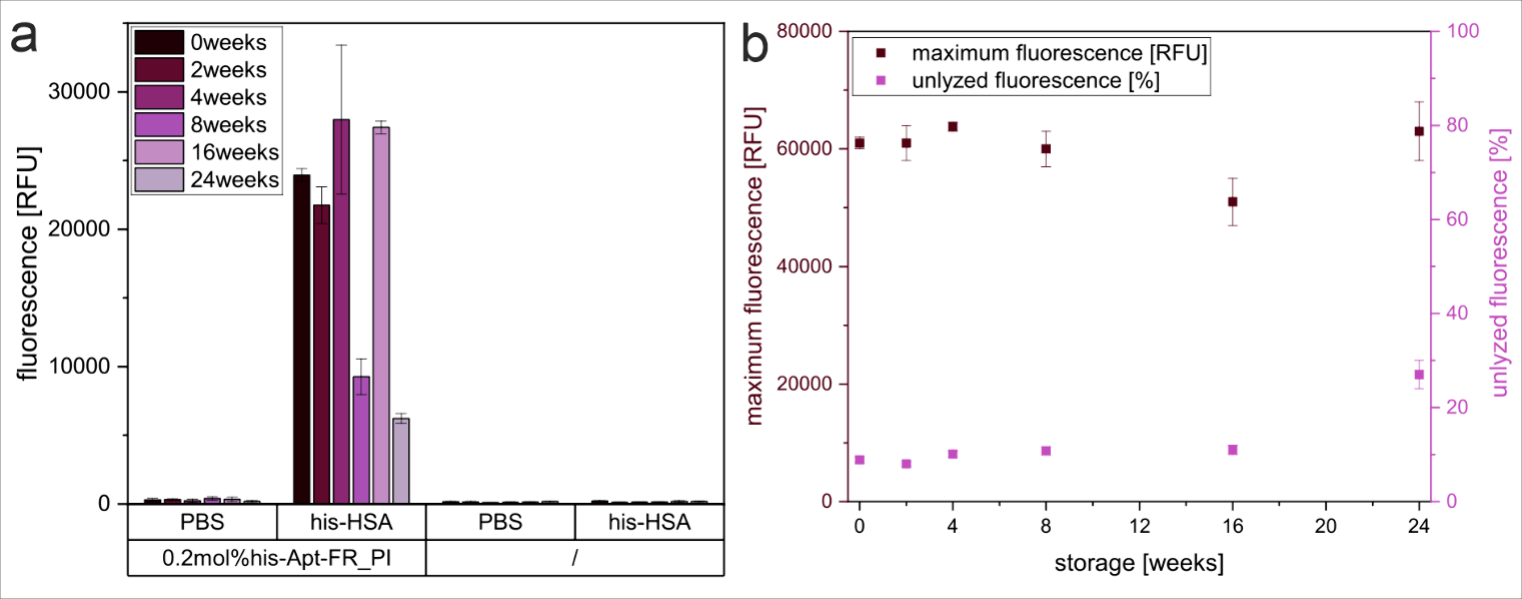
Storage stability of his-Apt-liposome conjugates, as derived from (a) fluorescence intensity over 24 weeks of liposomes modified with 0.2 mol % Chol-his-Apt-FR through post-insertion, stored at 4 °C or unmodified (/), immobilized in a Nunc MaxiSorp high-binding microplate coated with his-HSA (0 μg/mL or 2 μg/mL in PBS). *n* = 3. (b) maximum fluorescence and unlyzed fluorescence over 24 weeks of liposomes modified with 0.2 mol % Chol-his-Apt-FR through post-insertion, stored at 4 °C. *n* = 4.

### Application of Aptamer-Liposomes in Bioanalysis

#### Using His-Apt-Liposome Conjugates as a Tool for His-Tagged Protein Screening

The his-tag is a common modification of recombinant proteins, added as a fusion tag to enable affinity-based purification by immobilized metal ion affinity chromatography (IMAC), with the most established version being the Ni-NTA procedure.^57^ While the his-tag is added to the N- or C-terminus of the proteins, differences in its accessibility can occur due to protein folding, aggregation, or intramolecular interactions.^58,57^ However, this displays an essential factor to enable sufficient separation and purification of the proteins from lysates for downstream applications, like immobilization or functional studies.^59–62,57^ The his-Apt aptamers were initially developed as a his-tag masking probe for the selection of protein-specific aptamers.^66^ Here, their binding affinity to various recombinant his-tagged proteins in solution was first determined using microscale thermophoresis (MST). Therefore, binding of the 5′-Cy5-his-Apt to his-HSA containing a C-terminal poly his tag, and his-tagged RBD and ACE2, both containing a C-terminal combined Avi-his-tag, was investigated (Figure S6). Sabrowski et al. had carried out the same experiment using a hexa-his-peptide.^66^ As a negative control, an unrelated 80-nucleotide ssDNA sequence (Con1) was used. For his-HSA, a low dissociation constant (K_d_) value of (11 ± 6) nM was determined, suggesting strong binding between aptamer and his-HSA. In the case of his-RBD with its Avi-his_6_-tag, the K_d_ value = (3.2 ± 0.7) μM suggests weaker but existing binding. The decrease in binding affinity compared to his-HSA can most likely be explained by shielding of the his-moiety through the additional Avi-tag. Interestingly, his-ACE2 appeared not to bind to the aptamer, with the curve overlaying the control sequence and no reaching of significant response amplitudes. Subsequently, fully congruent results were found using the his-Apt-FR-liposome conjugates (Figure 4), demonstrating the full functionality of the conjugates. It is therefore suggested that the his-Apt-FR-tagged liposomes can serve as a fast and efficient tool for screening his-tagged proteins in a microtiter plate-based format. This provides a clear, semi-quantitative assessment of the accessibility of the his-tag and reveals advantages over the commonly used metallic nanoparticles in combination with Ni-NTA or Co-NTA, given the reliable applicability to crude supernatants, like bacterial cell lysate, without influencing or interfering with the conjugate performance and without the need for extensive optimization procedures (Figure S8).

**Figure 4:**
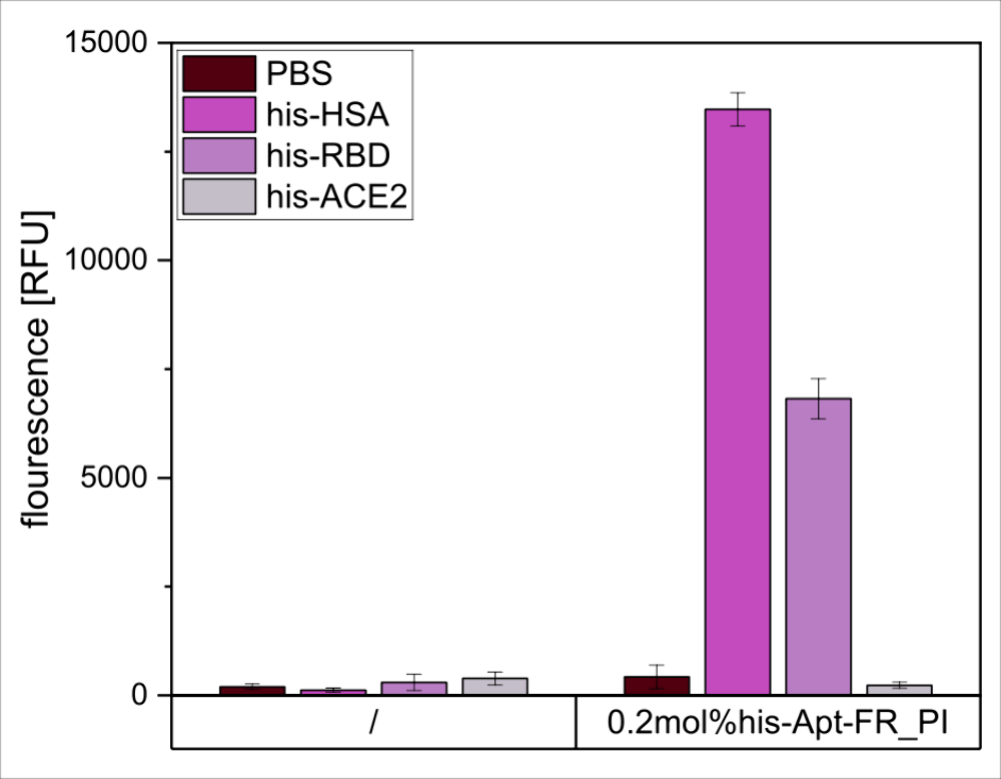
His-tag accessibility screening in recombinant proteins, presented through fluorescence intensity of liposomes modified with 0.2 mol % Chol-his-Apt-FR through post-insertion or unmodified (/), immobilized in a Nunc MaxiSorp high-binding microplate coated with his-HSA, his-RBD, or his-ACE2 (0 μg/mL or 2 μg/L in PBS). *n* = 3.

### Using Aptamers as Capture Ligand on the Liposome Surface

The his-Apt-FR aptamer was used as capture ligand to enable site-directed immobilization of his-tagged proteins on the liposome surface. Hence, especially with this aptamer, any recombinantly produced his-tagged protein can be immobilized on liposome surfaces through a very gentle process in which the aptamer functions as an anchor and additionally as a spacer between the liposome surface and the protein. Thereby, the need for additional processing or modifications, e.g., biotinylation or click chemistry groups, otherwise used for such site-directed strategies, can hence be avoided. In general, this technology could additionally be extended to any aptamer directed against other proteins or moieties. In our example, his-Apt-FR-liposomes were incubated with his-tag HSA in a dynamic concentration range from 25 ng/mL to 1000 ng/mL for 30 min. The successful coupling of HSA was demonstrated by its capturing the functionalized liposomes in an anti-HSA antibody-coated microtiter plate, with a low LOD of 5.7 ng/mL (Figure 5a). The same principle was proven by immobilizing his-tagged RBD on liposomes and capturing the complex via ACE2 immobilized in a microtiter plate in a dynamic range from 25–300 ng/mL, enabling excellent sensitivity with a LOD of 2.4 ng/mL. (Figure 5b). Furthermore, the aptamer-liposome approach was compared to the previously optimized covalent EDC/sNHS-mediated modification of liposomes with RBD. The amount of RBD used for these optimized 0.2 mol % RBD-coupled liposomes^26^ correlated to a RBD concentration of 50 ng/mL with the aptamer as the ligand. While at equal RBD amounts, similar fluorescence intensities were obtained, a nearly 2-fold increased intensity was achieved with an RBD concentration of 200 ng/mL, distinctly outperforming covalently modified liposomes. This highlights the efficiency of the here presented approach using the aptamer as a ligand for directed immobilization of his-tagged proteins without the need for complex modifications (e.g., biotinylation), while avoiding the risk of affecting the protein functionality, known for other coupling strategies,^64^ and thereby represents a compelling alternative.

**Figure 5:**
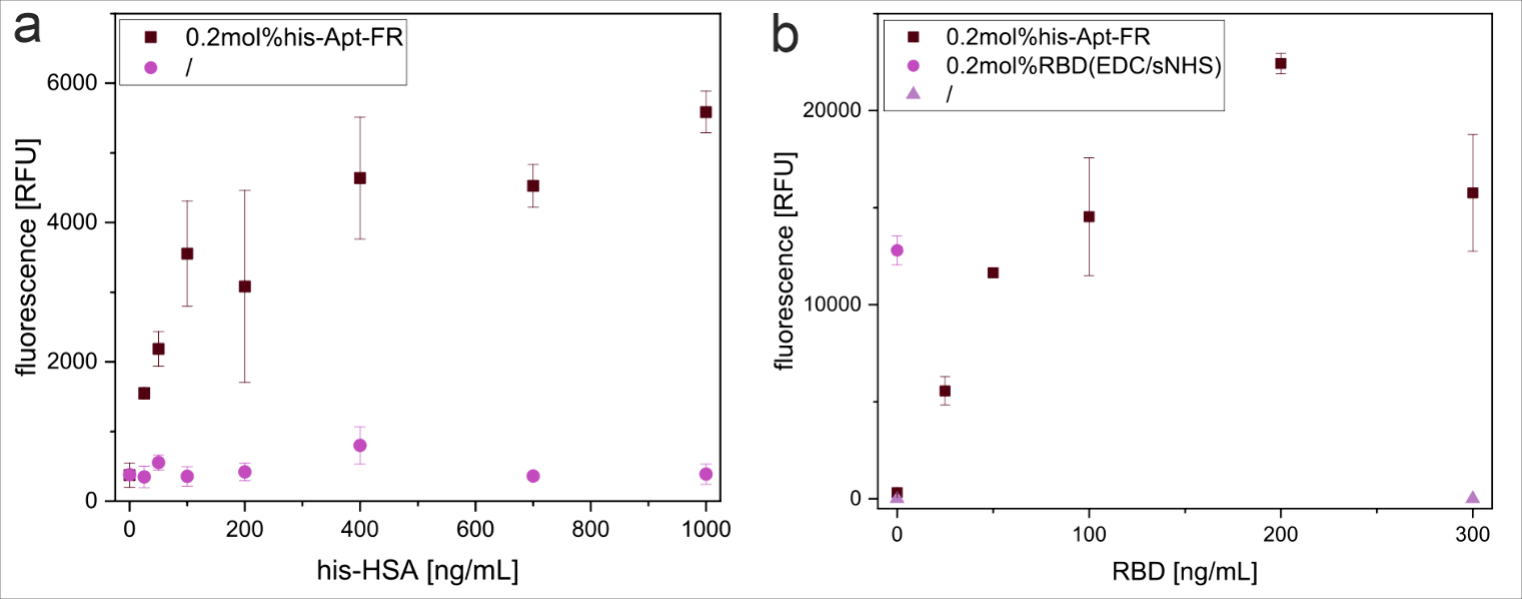
Capture of his-tagged proteins onto liposomes through his-Apt-FR, derived from the fluorescence intensity of liposomes modified with 0.2 mol % Chol-his-Apt-FR or uncoupled liposomes (/), immobilized together with (a) his-HSA (0 ng/mL–1000 ng/mL) in a Nunc MaxiSorp high-binding microplate coated with anti-HSA antibody (0 μg/mL or 5 μg/mL in PBS), or (b) his-RBD (0 ng/mL–300 ng/mL) as well as liposomes modified with 0.2 mol % RBD through EDC/sNHS in a Biomat streptavidin plate coated with ACE2-biotin (0 μg/mL or 2 μg/mL in PBS); with 30 min pre-incubation of the liposomes and his-proteins at RT. *n* = 3.

### Development of Dual-Tag Liposomes for Bioassays

For guaranteeing repeatability and reliability in bioassays, the implementation of control systems is required.^65^ As a progressive approach, which allows for universal internal control in liposome-based bioassays without the need to apply external standards or reference tests, dual-tag liposomes were developed. These liposomes contain both the biorecognition element of interest and the his-Apt-FR aptamer as control tag. Based on the assay format developed by Streif et al.^26^ for the detection of neutralizing SARS-CoV-2 antibodies using RBD-modified liposomes and ACE2, dual-tag liposomes containing RBD and his-Apt-FR simultaneously were prepared. RBD was covalently attached to the liposomes via EDC/sNHs, Chol-his-Apt-FR was post-inserted. The stability of the liposomes, as well as the functionality of both surface components, could be preserved at high levels (Figure 6a). By precisely finetuning the ratio to 0.1 mol % RBD and 0.2 mol % Chol-his-Apt-FR, maximum results were achieved, retaining 80% of theoretical binding ability for RBD and 100% for the aptamer in the optimized balance of the RBD and aptamer ratio (Figure S7). This enables reliable monitoring of assay performance through normalization on the aptamer-his-HSA binding under the integration of environmental factors influencing the general assay performance. This system is thereby applicable to complex assay types, shown by the neutralization of RBD with anti-RBD antibodies, where the behavior of RBD on the dual liposomes remained consistent with that of RBD control liposomes solely modified with 0.1 mol % RBD (Figure 6b). Highly comparable EC50 values of (0.067 ± 0.005) μg/mL (dual liposomes) and (0.08 ± 0.01) μg/mL (RBD liposomes) confirm the continuous functionality of RBD, while the aptamer–his–tag binding remained constant and unaffected throughout the anti-RBD antibody concentration range tested. The latter proves the reliable applicability as an internal control system.

**Figure 6:**
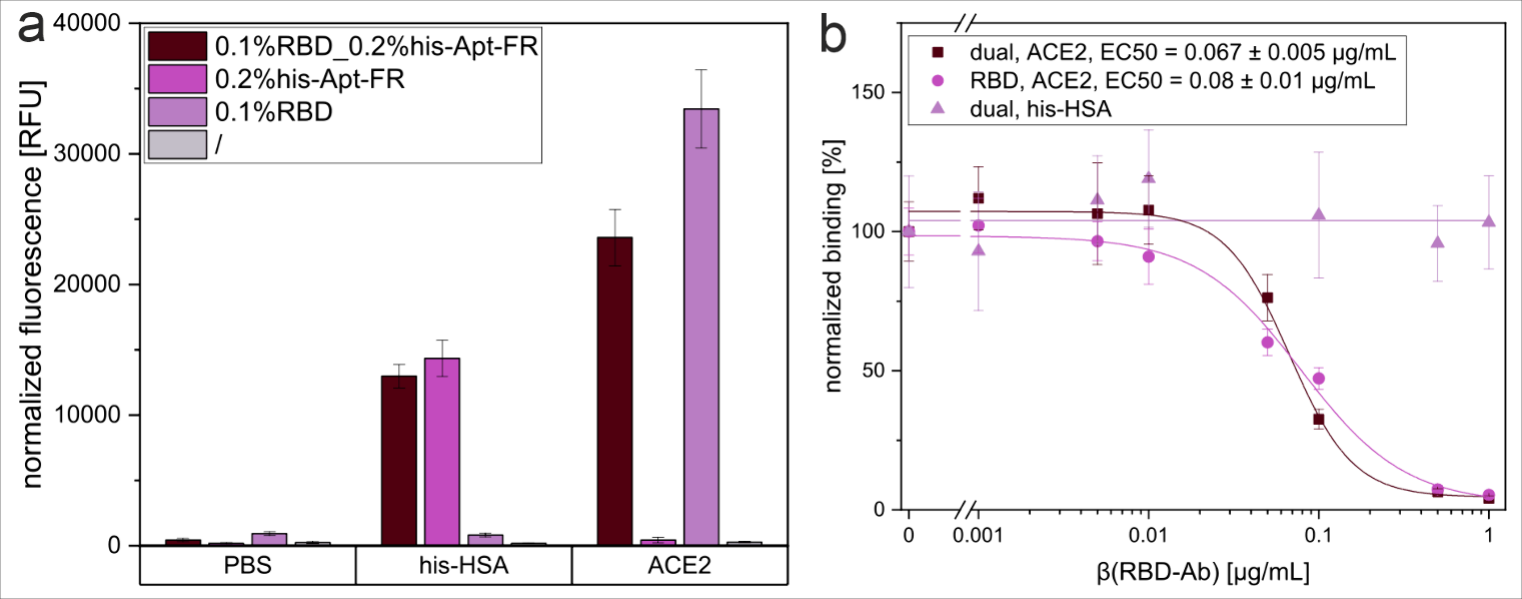
Functionality of the dual-liposome internal control setup, displayed by (a) normalized fluorescence intensity of liposomes modified simultaneously with 0.1 mol % RBD and 0.2 mol % Chol-his-Apt-FR, or only 0.1 mol % RBD or 0.2 mol % Chol-his-Apt-FR, or unmodified (/), immobilized in a Nunc MaxiSorp high-binding microplate coated with ACE2 or his-HSA (0 μg/mL or 2 μg/mL in PBS). (b) normalized binding intensity of liposomes modified with 0.1 mol % RBD and 0.2 mol % Chol-his-Apt-FR or only 0.1 mol % RBD, immobilized in a Biomat streptavidin plate coated with ACE2-biotin or his-HSA-biotin (2 μg/mL in PBS) after pre-incubation with anti-RBD antibodies (0.001–1 μg/mL) for 2 h at RT. *n* = 3.

## Conclusion

The application of aptamer-liposome conjugates in bioassays is of rising interest based on the combination of the outstanding signal amplification characteristics of liposomes^25,19^ with the excellent properties of aptamers as a biorecognition element.^82,14,81,13,15^ In this work, functional aptamer-liposome conjugates were prepared, investigating the anti-his-tag aptamer his-Apt and its truncated version his-Apt-FR, as a versatile multipurpose tool for bioassays. Crucial design criteria include the preservation of the relevant aptamer structure and the consistent integrity of the liposomes, which were fulfilled by optimizing the coupling processes and identifying suitable buffer compositions. Since the chosen aptamer does not contain a structure-stabilizing G-quadruplex, we suggest that the optimized conditions may be applicable to a broad range of aptamers, including those with weak secondary structure motifs.

The his-tag-aptamer-liposome conjugates furthermore allow for a broad applicability as a tool for his-tag screening, as an anchor for non-covalent coupling, or as an internal control system. His-tag screening will significantly improve protein purification, as the conditions under which the his-tag accessibility is optimal can be rapidly examined. Furthermore, considering the known drawbacks of metal-based IMAC protein purification strategies,^61,83^ future studies will investigate the use of his-Apt-modified beads and resins for protein purification from lysates.

The use of the his-tag aptamer as a ligand for gentle coupling of recombinant proteins onto the liposome surface adds a simple method for surface modifications and hence augments the standard portfolio of adsorptive, covalent, and streptavidin-mediated strategies. Since recombinant proteins often bear a his-tag, the aptamer ligand method is indeed superior to the other coupling methods, since no additional recombinant or chemical modification of the proteins is needed. Hence, additional processing steps, which inevitably result in a loss in yield and/or protein functionality, can be avoided. Finally, aptamers are seamlessly integrated as secondary ligands in protein-modified liposomes, without affecting the liposome characteristics. Functionality of aptamer and protein is maintained, liposome integrity easily ensured, and therefore a multitude of applications foreseeable, including those as an internal control. The here presented aptamer-liposome conjugates are hence a very versatile platform technology that will have a big impact on protein-based delivery and bioassays.

## Supplementary Material



## Notes

Additional information regarding liposome-aptamer coupling through EDC/sNHS or the membrane anchor method, as well as data on the conjugate characteristics for all coupling strategies. Optimization procedures for conjugate preparation via post-insertion or non-covalently. Calculated structures of the aptamers his-Apt and his-Apt-FR, and MST measurements with his-HSA, his-RBD, and his-ACE2. Optimization process of functional group ratios for dual liposomes. (PDF)
