# Supplementary material for: His-Tag-Aptamer-Liposome-Conjugates as a Multipurpose Tool for Recombinant Protein Screening and Protein-Based Bioassays
Source: Anal Chem. 2026 Apr 24;98(18):13609–19. doi: 10.1021/acs.analchem.6c00320 (PMC13177289; doi:10.1021/acs.analchem.6c00320)
Supplement: Supplementary file 1 [file ac6c00320_si_001.pdf]

Supporting Information

His-Tag-Aptamer-Liposome-Conjugates as a Multipurpose Tool for Recombinant Protein Screening and Protein-Based Bioassays

Pia Schraml[a], Florian K. Blaser[ab], Simon Streif[a], Nico Dreymann[b], Axel Duerkop[a], and Antje J. Baeumner[ab].*

[a]University of Regensburg, Faculty of Chemistry and Pharmacy, Institute of Analytical Chemistry, Chemo- and Biosensors, Universitätsstraße 31, 93053, Regensburg, Germany

[b]Fraunhofer Institute for Cell Therapy and Immunology, Branch Bioanalytics and Bioprocesses (IZI-BB), Am Mühlenberg 13, 14476, Potsdam-Golm, Germany

* antje.baeumner@ur.de

Table of contents

Calculated structures of the anti-his-tag aptamers his-Apt and his-Apt-FR: 3

Binding assay of aptamer-liposome conjugates prepared through the membrane anchor method: 4

Characterization data and binding assays of conjugates prepared with EDC/sNHS covalent coupling: 5

Characterization data and binding assays for the optimization of incubation temperature and aptamer content for post-insertion: 7

Binding assay for the optimization of aptamer content for biotin-streptavidin non-covalent coupling: 9

Microscale thermophoresis measurements for determination of aptamer – his-tag-protein binding abilities: 10

Binding assays for optimization of the RBD to his-Apt-FR ratio for preparation of dual-modified liposomes: 11

Binding assay for a functionality assessment of his-Apt-liposome conjugates in bacterial cell lysate: 12

Calculated structures of the anti-his-tag aptamers his-Apt and his-Apt-FR:

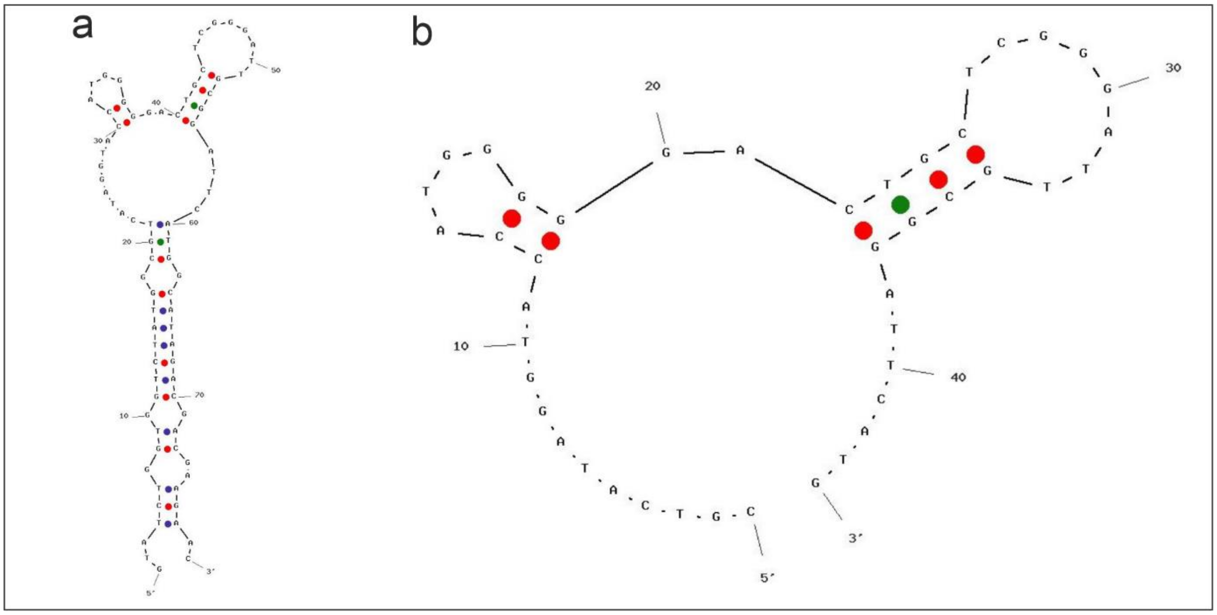

Figure S1. Calculated structure of the (a) his-Apt and (b) his-Apt-FR aptamer at ionic conditions of c (Na^+) = 140 mM, c (Mg^{2+}) = 5 mM, and 25 °C ambient temperature, according to UNAFold.

Binding assay of aptamer-liposome conjugates prepared through the membrane anchor method:

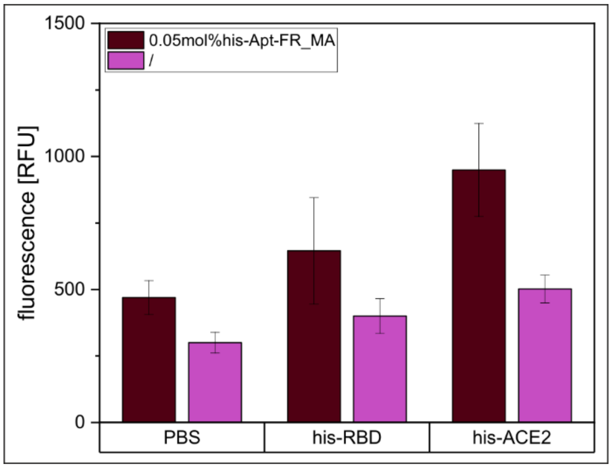

Figure S2. Fluorescence intensity of liposomes containing 0.05 mol% Chol-his-Apt-FR prepared through the membrane anchor method (MA), or unmodified liposomes (/), immobilized in a Nunc MaxiSorp high-binding microplate coated with his-RBD, or his-ACE2 (0 µg/mL or 1 µg/mL in PBS). n = 3.

Characterization data and binding assays of conjugates prepared with EDC/sNHS covalent coupling:

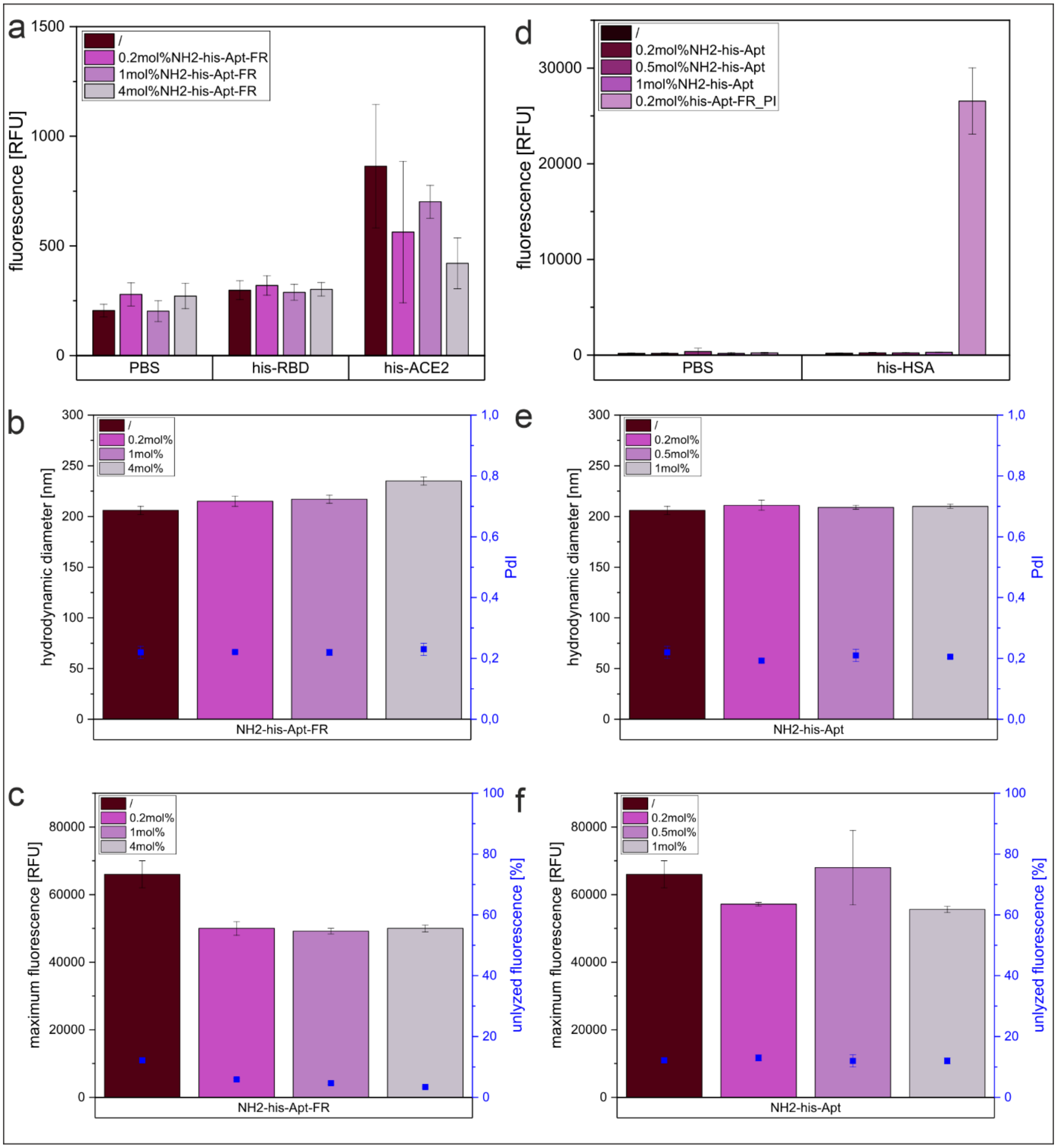

Figure S3. (a,d) Fluorescence intensity of liposomes modified with 0.2 – 4 mol% NH2-his-Apt-FR (a) or 0.2 – 1 mol% NH2-his-Apt (b) through covalent EDC/sNHS coupling, as well as unmodified liposomes (/) or post-insertion positive control liposomes (PI), immobilized in a Nunc MaxiSorp high-binding microplate coated with his-RBD, his-ACE2 (a) or his-HSA (b) (0 µg/mL,

or 2 µg/mL in PBS). n = 3. (b,e) hydrodynamic diameter and PdI, and (c,f) maximum fluorescence as well as unlysed fluorescence intensity of liposomes modified with 0.2 – 4 mol% NH2-his-Apt-FR (b,c) or 0.2 – 1 mol% NH2-his-Apt (e,f) through EDC/sNHS. n = 3 – 4.

Characterization data and binding assays for the optimization of incubation temperature and aptamer content for post-insertion:

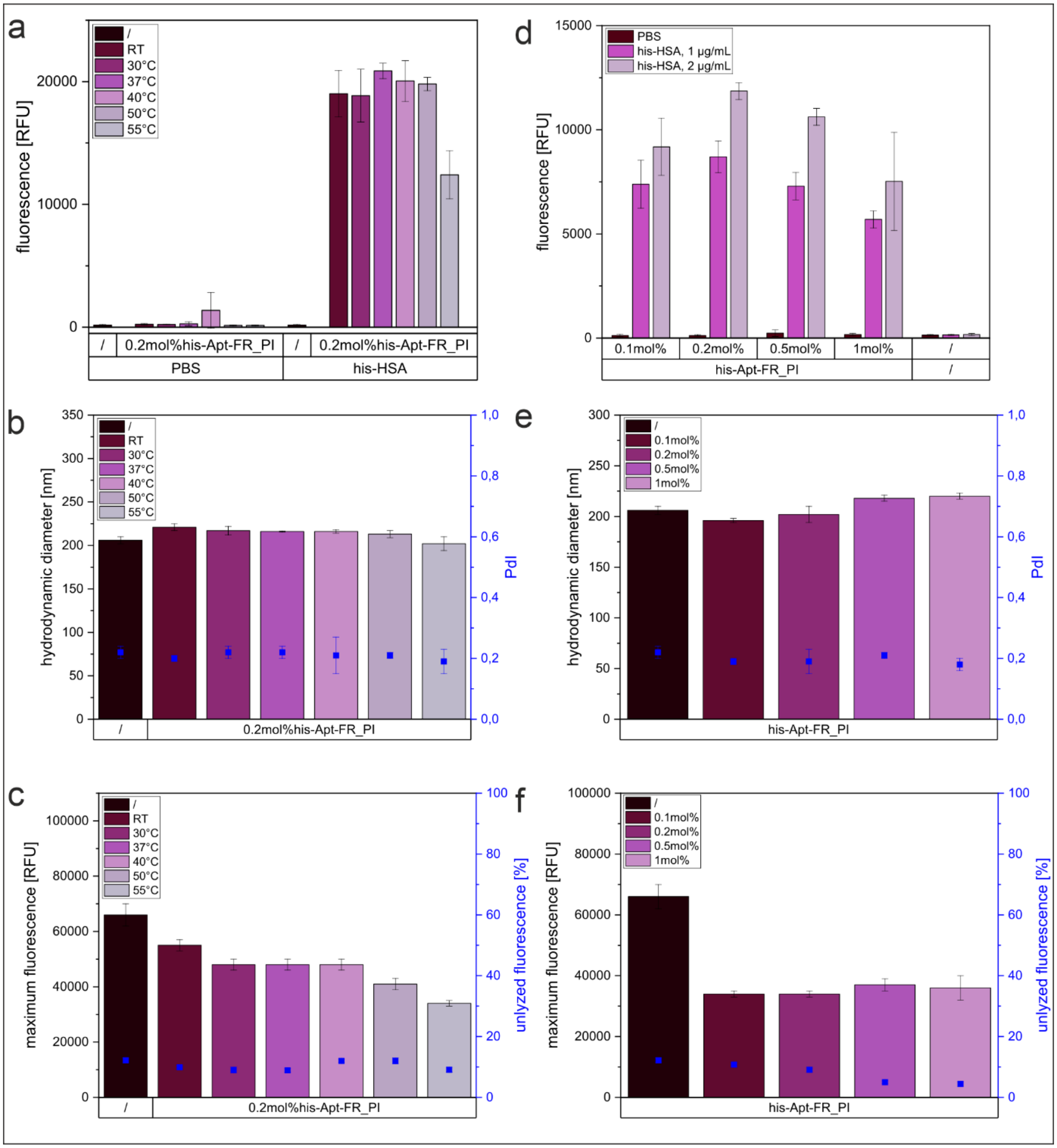

Figure S4. (a,d) Fluorescence intensity of liposomes modified with 0.1 – 1 mol% Chol-his-Apt-FR at 55 °C (a) or 0.2 mol% Chol-his-Apt-FR at RT – 55 °C (b) through post-insertion (PI), as well as unmodified liposomes (/), immobilized in a Nunc MaxiSorp high-binding microplate coated with his-HSA (0 µg/mL, 1 µg/mL, or 2 µg/mL in PBS). n = 3. (b,e) hydrodynamic diameter

and PdI, and (c,f) maximum fluorescence as well as unlysed fluorescence intensity of liposomes modified with 0.1 – 1 mol% Chol-his-Apt-FR at 55 °C (b,c) or 0.2 mol% Chol-his-Apt-FR at RT – 55 °C (e,f) through post-insertion. n = 3 – 4.

Binding assay for the optimization of aptamer content for biotin-streptavidin non-covalent coupling:

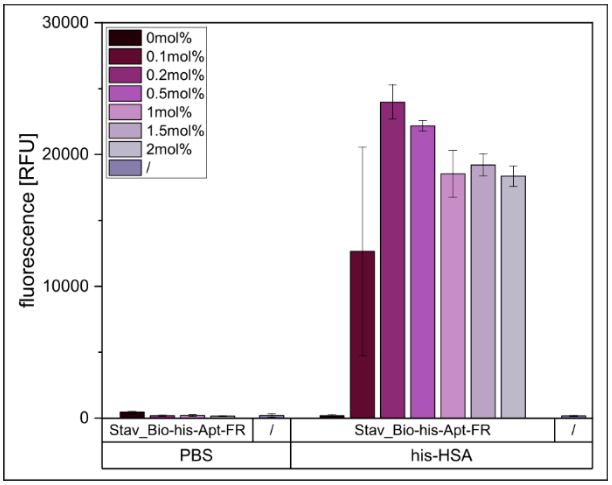

Figure S5: Fluorescence intensity of liposomes modified with 1.38 mol% stav or unmodified liposomes, immobilized together with biotin-his-Apt-FR (0 - 2 mol%) in a Nunc MaxiSorp high-binding microplate coated with his-HSA (2 µg/mL in PBS, 100 µL). n = 3.

Microscale thermophoresis measurements for determination of aptamer – his-tag-protein binding abilities:

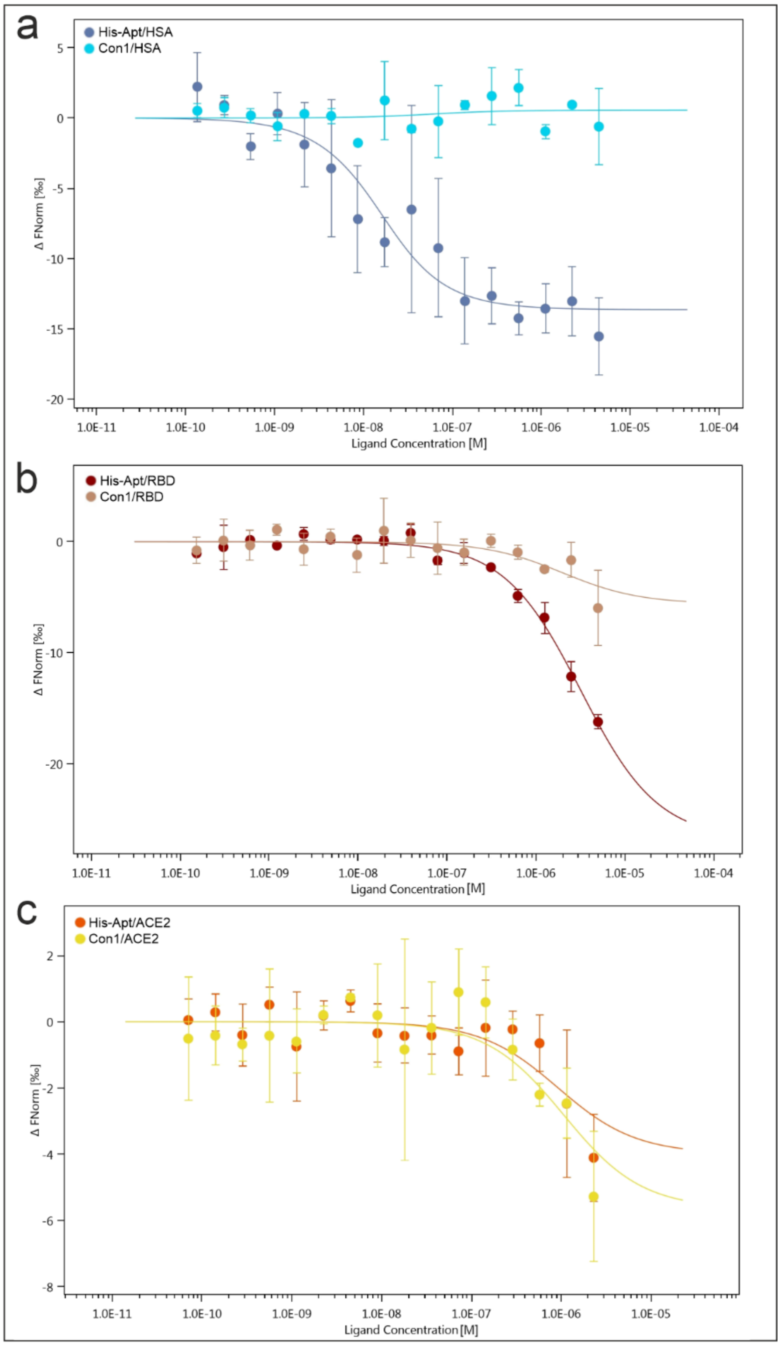

Figure S6. Microscale thermophoresis measurement of the 5'-Cy5-his-Apt aptamer (dark, 10 nM) or 5'-Cy5-Con1 aptamer (Con1, light, 10 nM) with (a) his-HSA, (b) his-RBD, and (c) his-ACE2 (70 pm – 5 μM). n= 2-3.

Binding assays for optimization of the RBD to his-Apt-FR ratio for preparation of dual-modified liposomes:

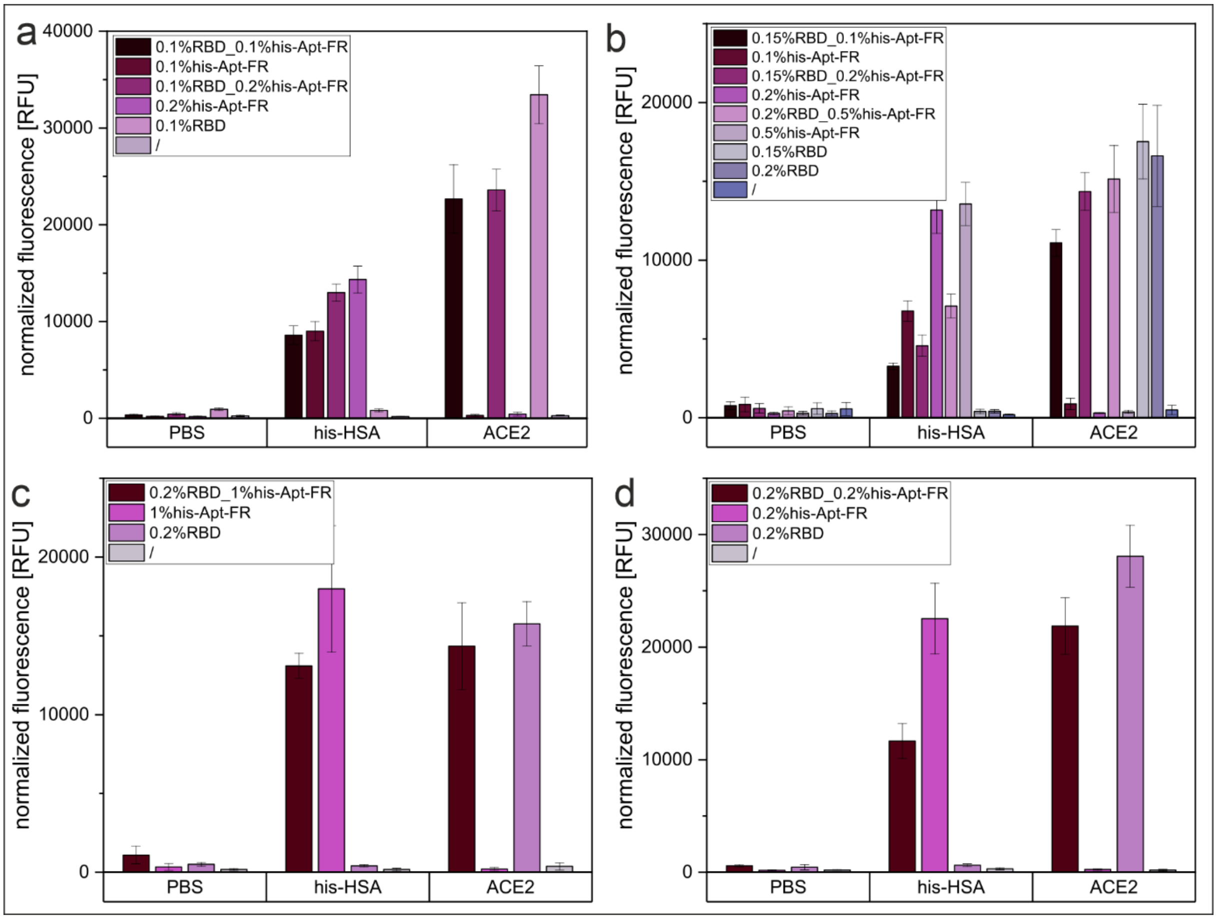

Figure S7. normalized fluorescence intensity of liposomes modified simultaneously with RBD and Chol-his-Apt-FR at varying concentrations of (a) 0.1 mol% RBD and 0.1 – 0.2 mol% Chol-his-Apt-FR, (b) 0.15 – 0.2 mol% RBD and 0.1 – 0.5 mol% Chol-his-Apt-FR, (c) 0.2 mol% RBD and 1 mol% Chol-his-Apt-FR (d) 0.2 mol% RBD and 0.2 mol% Chol-his-Apt-FR, or only either RBD or Chol-his-Apt-FR at the respective concentrations, immobilized in a Nunc MaxiSorp high-binding microplate coated with ACE2 or his-HSA (0 or 2 µg/mL in PBS). n = 3.

Binding assay for a functionality assessment of his-Apt-liposome conjugates in bacterial cell lysate:

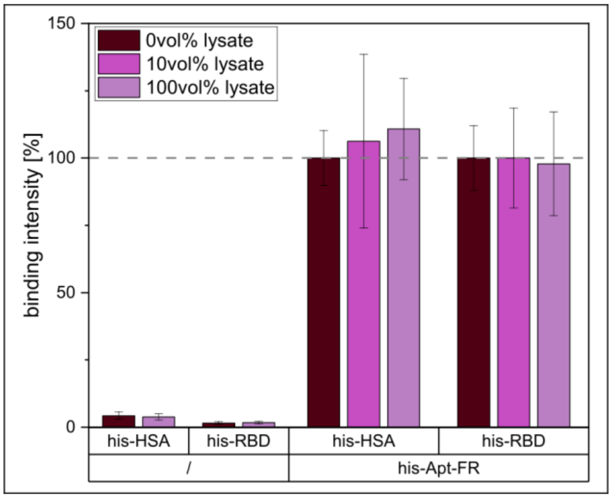

Figure S8. fluorescence intensity of liposomes modified with 0.2 mol% Chol-his-Apt-FR, incubated with 0 vol%, 10 vol%, and 100 vol% *E. coli* cell lysate in a Nunc MaxiSorp high-binding microplate coated with his-HSA or his-RBD (2 µg/mL in PBS). n = 3.
